# Oxaliplatin plus S-1 with intraperitoneal paclitaxel for the treatment of Chinese advanced gastric cancer with peritoneal metastases

**DOI:** 10.1186/s12885-021-09027-5

**Published:** 2021-12-18

**Authors:** Min Shi, Zhongyin Yang, Sheng Lu, Wentao Liu, Zhentian Ni, Xuexin Yao, Zichen Hua, Runhua Feng, Yanan Zheng, Zhenqiang Wang, Birendra Kumar Sah, Mingmin Chen, Zhenglun Zhu, Changyu He, Chen Li, Jun Zhang, Chao Yan, Min Yan, Zhenggang Zhu

**Affiliations:** 1 Department of Oncology, Ruijin Hospital, Shanghai Jiao Tong University School of Medicine, No. 197 Ruijin er Road, Shanghai, 200025 China; 2 Department of General Surgery, Shanghai Key Laboratory of Gastric Neoplasms, Shanghai Institute of Digestive Surgery, Ruijin Hospital, Shanghai Jiao Tong University School of Medicine, No. 197 Ruijin er Road, Shanghai, 200025 China; 3 State Key Laboratory of Oncogenes and Related Genes, Shanghai Jiao Tong University School of Medicine, Shanghai, 200025 China

**Keywords:** Gastric cancer, Peritoneal metastasis, Oxaliplatin, S-1, Intraperitoneal paclitaxel

## Abstract

**Background:**

In this study, we tried to access the efficacy and safety of oxaliplatin plus S-1 with intraperitoneal paclitaxel (PTX) for the treatment of Chinese advanced gastric cancer with peritoneal metastases.

**Patients and methods:**

Thirty patients diagnosed with advanced gastric cancer underwent laparoscopic exploration and were enrolled when macroscopic disseminated metastases (P1) were confirmed. PTX was diluted in 1 l of normal saline and IP administered through peritoneal port at an initial dose of 40 mg/m^2^ over 1 h on day1,8, respectively. Oxaliplatin was administered intravenously at an initial dose of 100 mg/m^2^ on day1, and S-1 was administered orally at an initial dose of 80 mg/m^2^ for 14 days followed by 7 days rest, repeated by every 3 weeks.

**Results:**

Of all these 30 patients, the median number of cycles was 6 (range 2–16) due to the limitation of hematotoxicity and peripheral neuropathy by oxaliplatin. There were 11 (36.7%) patients received conversion surgery. The median progression free survival (PFS) was 6.6 months (95% CI = 4.7–8.5 months) and the median overall survival (OS) was 15.1 months (95% CI = 12.4–17.8 months). The grade 3–4 hematological toxicities were leucopenia (23.3%), neutropenia (23.3%), anemia (16.7%), and thrombocytopenia (20%), respectively. The grade 3–4 non-hematological toxicities were tolerated, most of which were peripheral sensory neuropathy (40%) due to oxaliplatin, diarrhea (20%), nausea and vomiting (26.7%).

**Conclusions:**

SOX+ip PTX regimen was effective in advanced gastric cancer with peritoneal metastasis. Survival time was significantly prolonged by conversion surgery. Grade 3–4 toxicities were uncommon. Large scale clinical trial is necessary to get more evidence to identify its efficacy.

**Trail registration:**

ChiCTR, ChiCTR-IIR-16009802. Registered 9 November 2016,

## Background

According to the global cancer statistics, there will be nearly 18.1 million new cancer cases in 2018, and estimated that there will be 9.6 million cases died of cancer related diseases. Among them, gastric cancer ranks fifth in all cancer incidence (5.7%) and mortality (8.2%) [1]. As China has not yet formed a perfect gastrointestinal tumor screening system, the early diagnosis rate needs to be improved [2], and most patients are in the advanced stage when diagnosed, even with distant metastasis [3, 4]. It was reported that nearly 20% of gastric cancer patients were diagnosed with peritoneal metastasis (PM) before or during operation [5], while more than 50% of gastric cancer patients will have peritoneal metastasis in the future even after radical resection. Therefore, peritoneal metastasis is the first cause of death in patients with gastric cancer, whether resectable or unresectable [6–8].

“Seed soil” is the main theory to explain peritoneal metastasis. As far as the source of seeds is concerned, as the primary lesion of gastrointestinal tumor penetrates the serosal layer and infiltrates into the surrounding tissues or organs, the tumor cells fall off and form peritoneal free cancer cells [9, 10]. Improper operation may also increase the risk of iatrogenic spread, such as the squeezing of tumor cells by the operator or the entrance of the abdominal cavity through the amputated lymph vessels and lymph vessels. Secondly, the rough separation surface caused by surgical detachment can form “soil”. Finally, a large number of cytokines released during wound healing and angiogenesis factors can be regarded as “nourishment”, which together constitute the microenvironment suitable for the formation of peritoneal metastasis.

According to the Chicago consensus on peritoneal metastasis in 2020 [11], platinum and fluorouracil based regimens, including FOLFOX (oxaliplatin + calcium folinate + fluorouracil), XELOX (oxaliplatin + capecitabine), FLOT (docetaxel + calcium folinate + fluorouracil), ECF (epirubicin + cisplatin + fluorouracil) and SP (cisplatin + S-1), can be considered as the first-line chemotherapy for gastric cancer patients with peritoneal metastasis [12–17]. However, there is no clear evidence of molecular targeted drugs and immunotherapy drugs in peritoneal metastasis of gastric cancer. Due to the existence of blood peritoneal barrier [18], it is difficult to reach the effective therapeutic concentration of cytotoxic drugs given intravenously in the abdominal cavity. Systemic therapy is not always effective for peritoneal metastases, so intraperitoneal chemotherapy is naturally considered. However, due to the lack of drug permeability and uneven drug distribution, the response rate of peritoneal metastases to simple intraperitoneal administration is low [19, 20]. Therefore, scholars have proposed intraperitoneal combined with intravenous and oral chemotherapy, in order to improve the control of peritoneal metastasis by combining the advantages of local and systemic treatment [21, 22].

One hundred and eighty gastric cancer patients with peritoneal metastasis were enrolled in the Phoenix-GC study [23] carried out by Kitayama et al., Tokyo University, Japan. The experimental group was treated with paclitaxel intraperitoneal (IP-PTX, 20 mg/m^2^) chemotherapy combined with paclitaxel intravenous (IV-PTX) chemotherapy combined with oral S-1, while the control group was treated with intravenous infusion of cisplatin together with oral S-1, but the primary endpoint OS (overall survival) marginally failed to meet the predefined level of significance. Thus, Kitayama et al. carried out a phase I/II study [24], which combined IP-PTX (40 mg/m^2^) with systemic S-1/oxaliplatin (SOX) as induction therapy for GC patients with peritoneal metastasis.

In this study, we evaluated the efficacy and toxicity of intraperitoneal paclitaxel with systemic S-1 plus oxaliplatin in Chinese gastric cancer patients with peritoneal metastasis.

## Methods

### Study populations

This was a single-center, prospective study. Those patients whom diagnosed with advanced gastric cancer upon laparoscopic exploration were enrolled if macroscopic disseminated metastases (P1) was confirmed. The main eligibility criteria included: 1) Age from 18–75 years; 2) Adenocarcinoma was confirmed and classified as metastatic gastric cancer; 3) Chemotherapy, radiotherapy, target therapy, and immunotherapy were never used for those patients; 4) At least one measurable lesion should be provided for assessment as peritoneal metastasis by computed tomography (CT) scan or laparoscopy; 5) Good performance status with the Eastern Cooperative Oncology Group (ECOG) score of 0–1; 6) Proper bone marrow, liver and kidney function; 7) Survival period over 3 months. Exclusion criteria included: 1) Other distant organ metastasis; 2) Brain metastasis; 3) Pregnancy or breastfeeding; 3) Intestinal obstruction; 4) Symptomatic pulmonary fibrosis; 5) Concurrent malignancy or other uncontrolled severe diseases. This study was approved by the Ruijin Hospital Ethics committee of Shanghai Jiaotong University School of Medicine. Informed consent was obtained from all patients before registration in accordance with the Declaration of Helsinki. This study was registered in China Clinical Trial Registry (ChiCTR-IIR-16009802). This study was carried out by Department of Gastrointestinal Surgery and Oncology at Ruijin Hospital, Shanghai Jiaotong University School of Medicine.

### Treatments

When laparoscopic exploration was carried out, peritoneal port was placed in the subcutaneous space of the lower abdomen with a catheter placed in the pelvic cavity and peritoneal cancer index (PCI) was determined, which was quantitatively described the distribution of intraperitoneal tumors. PTX was diluted in 1 Litre of normal saline, then, IP was administered through the peritoneal port at an initial dose of 40 mg/m^2^ over 1 h on day1,8, respectively. Oxaliplatin was carried out intravenously at an initial dose of 100 mg/m^2^ on day1, and S-1 was carried out orally at an initial dose of 80 mg/m^2^ for 14 days followed by 7 days rest, and repeated for 3 weeks. When patients have responded from this combined regimen, laparoscopic exploration could be performed again to confirm whether macroscopic disseminated metastases were disappeared. If so, radical gastrectomy would be conducted. This combined regimen was discontinued after 8 cycles, and IP-PTX plus S-1 were last until 1 year or until disease progression or unacceptable toxicities.

### Efficacy and toxicity evaluation

Peritoneal metastasis was diagnosed and evaluated upon laparoscopy exploration. Evaluation of tumor response was carried out every 3 cycles according to the response evaluation in solid tumors criteria (RECIST) version 1.1 [25]. The volume of malignant ascites reflected tumor response, and was evaluated by CT scan according to the guidelines by Japanese Classification of Gastric Carcinoma [26]. Toxicity was assessed after each cycle by using National Cancer Institute Common Toxicity Criteria for Adverse Events (NCI-CTCAE) version 5.0 [27]. Grade 3–4 hematological toxicities were leucopenia, neutropenia, anemia, and thrombocytopenia. Grade 3–4 non-hematological toxicities were peripheral sensory neuropathy, diarrhea, nausea and vomiting.

### Statistical analysis

Data were analyzed using SPSS software (version 17.0; SPSS, Chicago, IL, USA). The descriptive statistics were used for safety evaluation. Continuous endpoints were expressed as mean ± standard deviations (SDs), while discrete data were expressed as frequency and percentage distributions. Overall response rate (ORR) and disease control rate (DCR) and their two-sided 95% confidence interval (CI) were calculated. Kaplan-Meier survival curves were performed using GraphPad Prism software. Log-rank test was used to compare the differences between two groups. A value of p < 0.05 was considered statistically significant.

## Results

### Enrollment patient characteristics

A total of 30 gastric cancer patients with peritoneal metastasis were enrolled from July 1, 2017 to June 30, 2019. Baseline evaluation and clinicopathological features are presented in Table 1. All patients received laparoscopy exploration and PCI score was assessed.

**Table 1:** Baseline characteristics of enrolled patients

Characteristics	Total patients (%)	Conversion surgery (%)
	***n*** = 30	***n*** = 11
**Sex**		
Male	14 (46.7%)	5 (45.5%)
Female	16 (53.3%)	6 (54.5%)
**Age (years)**		
Median	51	41
Range	29–74	29–74
**ECOG score**		
0–1	28 (93.3%)	11 (100%)
2	2 (6.7%)	0 (0)
**Previous chemotherapy**		
Yes	7 (23.3%)	0 (0)
No	23 (76.7%)	11 (100%)
**Histological type**		
Poorly differentiated adenocarcinoma	22 (73.3%)	6 (54.5%)
Signet ring cell carcinoma	8 (26.7%)	5 (45.5%)
**Lauren’s type**		
Intestinal	11 (36.7%)	4 (36.7%)
Diffuse	19 (63.7%)	7 (63.7%)
**Extent of peritoneal metastases**		
P2	4 (13.3%)	2 (18.2%)
P3	26 (86.7%)	9 (81.8%)
**Ascites**		
Yes	23 (76.7%)	7 (63.6%)
No	7 (23.3%)	4 (36.4%)
**PCI score**		
0–9	5 (16.7%)	3 (27.3%)
10–19	8 (26.7%)	5 (45.4%)
20–39	17 (56.6%)	3 (27.3%)
**Ovary metastasis**		
Yes	7 (23.3%)	3 (27.3%)
No	23 (76.7%)	8 (72.7%)

### Conversion surgery and responses

Of all these 30 patients, the median number of cycles was 6 (range 2–16) due to the limitation of hematotoxicity and peripheral neuropathy by oxaliplatin. There were 11 (36.7%) patients received conversion surgery (Radical resection of R0, D2 lymph node dissection). The median cycles of chemotherapy before conversion surgery were 9 (range 6–16). The patients’ profiles and surgical results were summarized in Table 2. Combined resections of ovary were performed in three patients. After surgery, according to the tumor regression grade (TRG), TRG 1 or TRG 2 in resected primary tumors were observed in 3/11 (27.3%) and 5/11 (45.5%) patients, respectively. The 19 patients who did not undergo conversion surgery did not receive the second laparoscopic exploration because CT examination showed that they could not reach R0 radical resection. Postoperative recurred in 8 cases of patients with peritoneal metastasis, and 3 female patients were also had ovary metastasis.

**Table 2:** The patients’ profiles and surgical results

Clibical variables	Number of patients (%)
**Scope of gastrectomy**	
Total	7(63.6%)
Distal	4(36.4%)
**Lymph node dissection**	
D1	0
D2	11(100%)
**Radical degree of operation**	
R0	11(100%)
R1	0
R2	0
**Combined resection**	
Ovary	3(27.3%)
**T staging**	
ypT2	2(18.2%)
ypT4a	9(81.8%)
**N staging**	
ypN0	3(27.3%)
ypN1	3(27.3%)
ypN2	2(18.2%)
ypN3	3(27.3%)
**Tumor regression grade**	
TRG 1	2(18.2%)
TRG 2	5(45.4%)
TRG 3	4(36.4%)
**Postoperative complications**	
Abdominal infection	1(9%)

### Survival

**Fig. 1:**
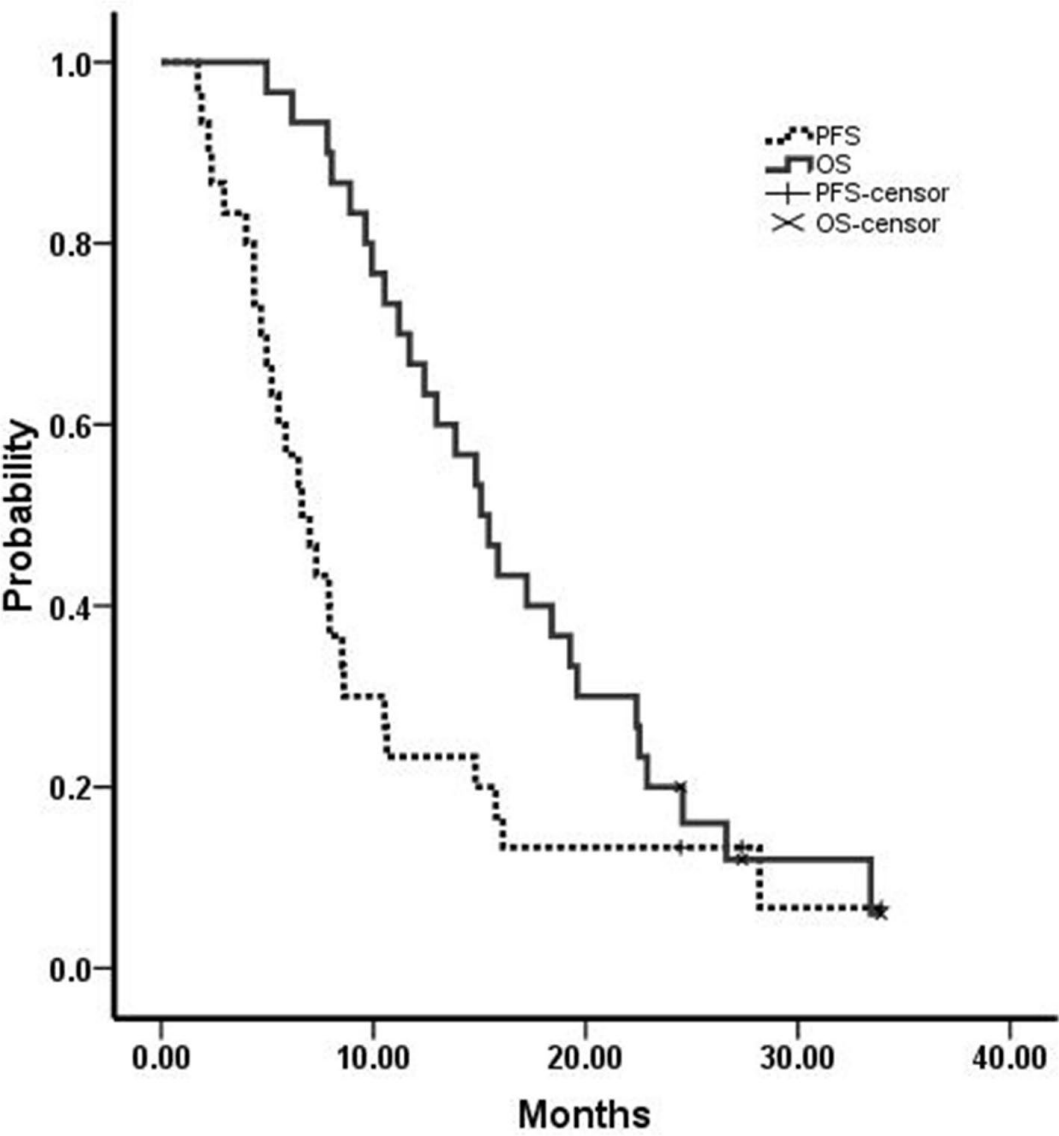
Kaplan-Meier curves of progression free survival (PFS) and overall survival (OS). Thirty advanced gastric cancer with peritoneal metastasis were treated with SOX+ip PTX regimen. The median PFS and OS were 6.6 months and 15.1 months respectively

**Fig. 2:**
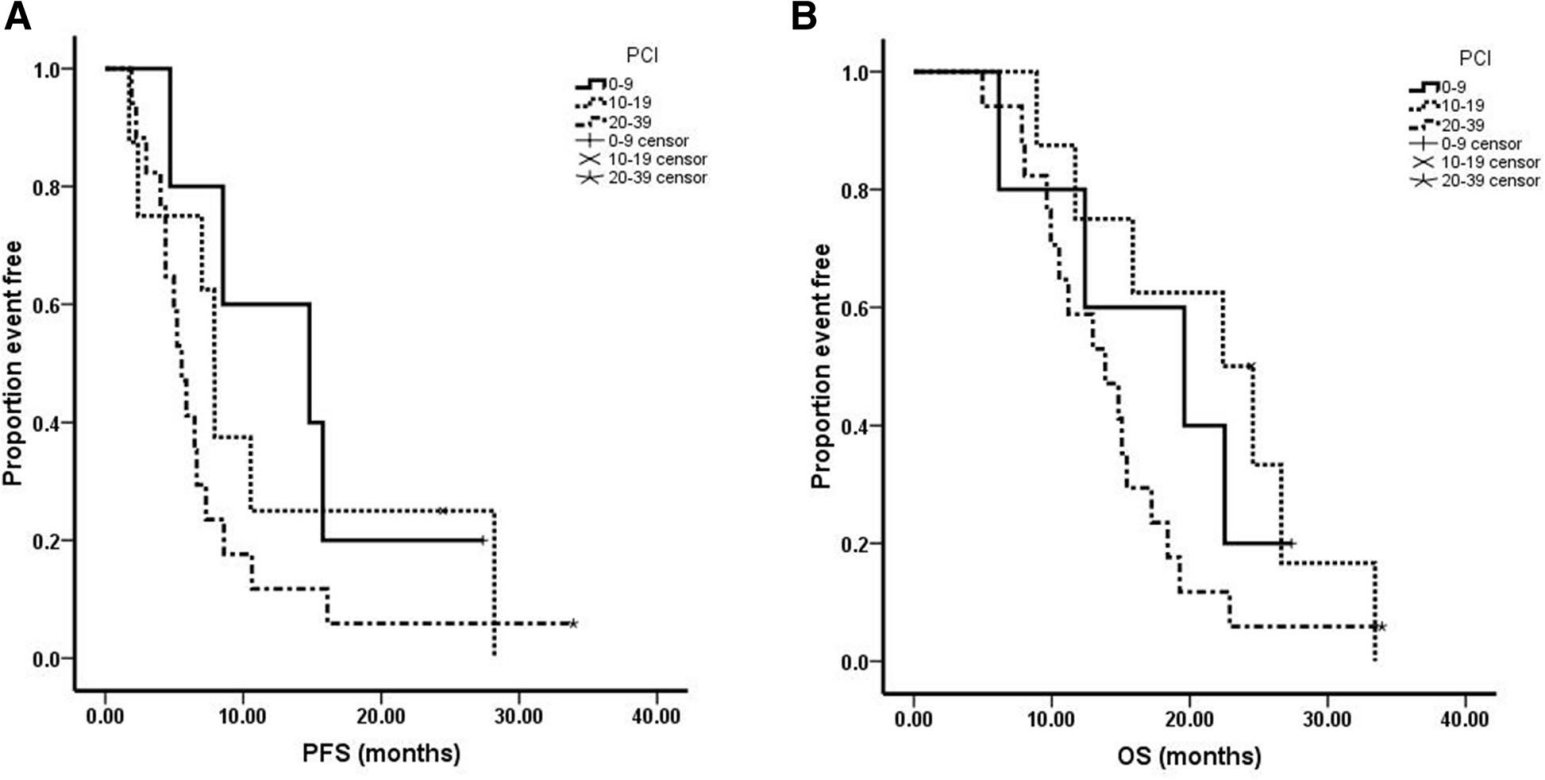
**A** The relationship between PCI scores and PFS (log-rank test). **B** The relationship between PCI scores and OS (log-rank test). There was a trend that those patients had shorter PFS and OS while PCI >20

**Fig. 3:**
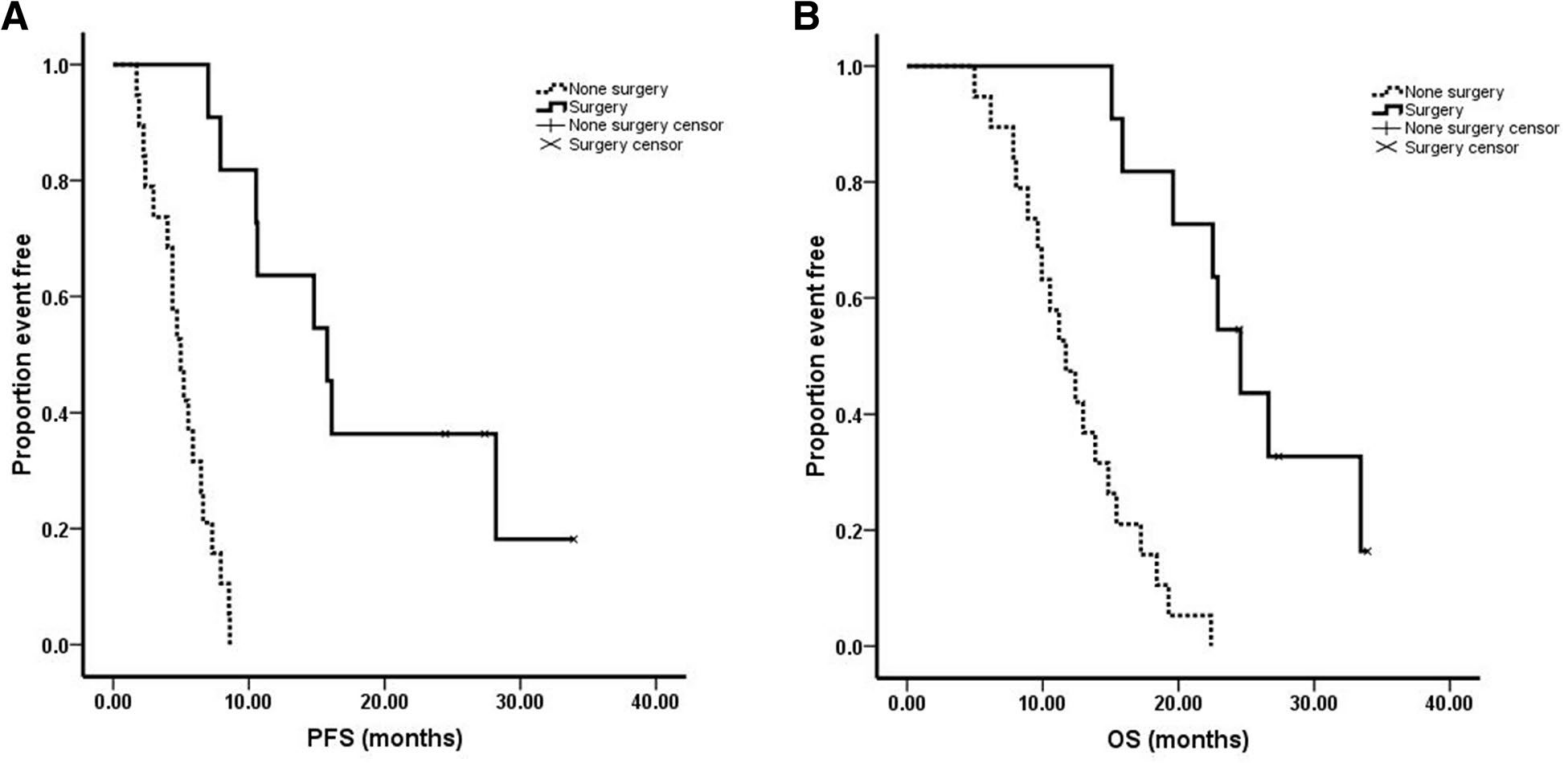
**A** The relationship between conversion surgery and PFS (log-rank test). **B** The relationship between conversion surgery and OS (log-rank test). Those patients whom received conversion surgery had longer PFS and OS

The median follow-up time was 15.3 months (range 5.0–33.9 months). The median progression free survival (PFS) was 6.6 months (95% CI = 4.7–8.5 months) and the median OS was 15.1 months (95% CI = 12.4–17.8 months, Fig. 1). From analyzing the PCI scores and survival among the 30 patients, we noticed that 1) although there were no statistics significance, there was a trend that those patients had shorter PFS (PCI 1–9: 14.8 months vs PCI 10–19: 7.9 months vs PCI: 20–39: 5.5 months, *P* = 0.266) and OS (PCI 1–9: 19.6 months vs PCI 10–19: 22.4 months vs PCI: 20–39: 13.9 months, *P* = 0.185) while PCI scores >20 (Fig. 2); 2) There was a significant difference between the patients whom received the conversion surgery or not, which means that those patients whom received conversion surgery had longer PFS (15.8 months vs 5.0 months, *P* < 0.01) and OS (24.6 months vs 11.7 months, *P* < 0.01) than those patients whom failed to received conversion surgery (Fig. 3).

### Toxicities

All 30 patients were evaluated for toxicity (Table 3). The grade 3–4 hematological toxicities were leucopenia (23.3%), neutropenia (23.3%), anemia (16.7%), and thrombocytopenia (20%), respectively. These patients received injections of granulocyte-colony stimulating factor (G-CSF) and recombinant human thrombopoietin (rhTPO), and all completed the treatment without interruption. The grade 3–4 non-hematological toxicities were tolerated, most of which were peripheral sensory neuropathy (40%) due to oxaliplatin, diarrhea (20%), nausea and vomiting (26.7%). No serious hepatorenal function abnormalities and treatment-related mortality were observed. The most common complication related to the peritoneal access device was peripump effusion and there is no needed for surgical intervention. There were no obstruction of the intraperitoneal catheter and infection of the access port were observed.

**Table 3:** Grade 3–4 toxicity hematological and non-hematological toxicities

	n	%
**Hematological toxicity**		
Leucopenia	7	23.30%
Neutropenia	7	23.30%
Anemia	5	16.70%
Thrombocytopenia	6	20%
**Non-hematological toxicity**		
Peripheral sensory neuropathy	12	40%
Diarrhea	6	20%
Nausea and vomiting	8	26.70%

## Discussion

The therapeutic strategy of GC with PM was neoadjuvant intraperitoneal and systemic chemotherapy (NIPS) followed by gastrectomy. It was reported that those GC patients with PM who were treated with NIPS using S-1 and IP administration of docetaxel (DTX) and cisplatin followed by cytoreductive surgery, the 1-year OS rate and median survival time (MST) were 67.4% and 15.0 months, respectively [28]. The patients who undergo gastrectomy after treatment with IP-PTX plus S-1/PTX, the 1-year OS and MST of were 73.3% and 30.5 months, respectively [29]. In our study, the conversion surgery rate after SOX+ ip PTX treatment was 36.7%, and the MST was 24.6 months (95% CI = 21.5–27.7 months). Meanwhile, grade 3–4 hematological/non- hematological toxicities were well tolerated. Kitayama et al. reported SOX+ip PTX regimens results, the conversion surgery rate was 45%, and the MST for those patients were 25.8 months [24]. Compared to our results, the conversion surgery rate was higher and the MST was similar. We analyzed the difference between the surgery rate, there may be two reasons: 1) All the surgery patients received R0 resection in our study and 2) the most patients in our study were diffuse type, which may be insensitive to oxaliplatin.

The understanding of peritoneal metastasis is gradually changing in the academic community, and now it is more likely to be defined as local lesions. Active and appropriate treatment can make some patients get a longer survival. Cytoreductive surgery (CRS) followed by hyperthermic intraperitoneal chemotherapy (HIPEC) is considered to benefit some patients with peritoneal metastasis of gastrointestinal tumors. For gastric cancer patients with PM, a phase III clinical study from China showed that the median OS of CRS followed by HIPEC group was longer than that of CRS alone group (11.0 months vs. 6.5 months, *P* < 0.05), suggesting that CRS combined with HIPEC can bring significant survival benefits to patients [30]. It is generally believed that patients with lower PCI value and better response to systemic therapy are potential beneficiaries. However, how much lower PCI value is suitable for surgery varies in different studies.

Although Phoenix-GC study failed to show significant improvement in OS, there are still some points to be circled, mainly including: setting of endpoint indicators, research quality control and operation, and stratification factors. Firstly, according to the results of SPIRIT study [31] which indicated that the OS was longer in patients with advanced gastric cancer treated with cisplatin plus S-1 than with S-1 alone, the researchers set the OS of the control group as 11 months, based on the 22 months OS of the study group in the previous phase II clinical study. As a pre-set indicator of phase III clinical study, it seems a bit optimistic and ambitious now. After all, it is difficult for advanced gastric cancer with PM to reach 22 months OS. In addition, from single center to multi center research, quality control and operation are also challenge. Secondly, retrospective analysis of data showed that in the control group, several patients violated the protocol and received intraperitoneal treatment, that is, the three patients who survived for more than three years in the control group (SP chemotherapy group) found in the study. Such data obviously affected the final outcome. Thirdly, no ascites, a small amount of ascites and moderate amount of ascites were taken as stratification factors in the study, but there was imbalance between the control group and the experimental group, which affected the final results; In the control group, there were 14 cases of small amount of ascites and 7 cases of moderate amount of ascites, while in the experimental group, there were 34 cases and 38 cases of study group respectively. It may be better to take the presence of ascites as the stratification factor.

Similarly, Phoenix-GC trial also has many highlights, including drug selection, route of administration, and drug delivery equipment. Firstly, the hydrophilic drugs used in the previous intraperitoneal chemotherapy, such as cisplatin and mitomycin C, are difficult to maintain a higher intraperitoneal concentration. Paclitaxel is a fat soluble drug, because of its poor water solubility, so all kinds of paclitaxel drugs are working hard on the type agent. The fat content of omentum, mesangium and other structures is high, local medication is conducive to drug distribution, and as a high molecular weight drug, it is conducive to maintain a high intraperitoneal drug concentration. But we need to think about the stability of taxanes in the temperature of HIPEC, and the selection of docetaxel and paclitaxel. Secondly, intraperitoneal local administration is conducive to maintaining the high concentration of local intraperitoneal. Compared with other hydrophilic chemotherapy drugs, taxanes cause less intra-abdominal adhesions and separation. Thus, multiple administration can be achieved without causing intra-abdominal adhesions and separation, affecting the uniform distribution of drugs. Thirdly, the way of infusion port is adopted to avoid the risk of repeated puncture, and laparoscopic catheterization is more intuitive.

Peritoneal metastasis is a difficult point in the prevention and treatment of gastrointestinal tumor, and it is also one of the hot spots in clinical and basic research. At present, the treatment is based on systemic chemotherapy, and the combination of surgery, intraperitoneal perfusion chemotherapy, HIPEC and other methods is an effective treatment for peritoneal metastasis of gastrointestinal tumor. However, breakthrough research results are still needed in the fields of risk prediction, assessment, prevention and treatment of peritoneal metastasis. How to enrich potential patients with peritoneal metastasis, how to determine the timing of conversion surgery, how to further optimize the existing treatment options, especially how to develop treatment options for patients after conversion surgery, still need to improve the research design and carry out prospective, randomized, controlled studies to solve the above clinical difficulties.

## Conclusions

SOX+ip PTX regimen was effective in advanced gastric cancer with PM. Survival time was significantly prolonged by conversion surgery. Our study indicated that most patients who received conversion surgery benefited from SOX plus IP-PTX regimens. Grade 3–4 toxicities were uncommon. Large phase III trial is necessary to obtain more evidence to identify its efficacy.

## Data Availability

The datasets analysed during the current study are available from the corresponding author on reasonable request.

## Abbreviations

IP-PTX: Paclitaxel intraperitoneal
IV-PTX: Paclitaxel intravenous
OS: Overall survival
CT: Computed tomography
ECOG: Eastern Cooperative Oncology Group
TRG: Tumor regression grade
PFS: Progression free survival
PCI: Peritoneal cancer index
RECIST: Response evaluation in solid tumors criteria
NCI-CTCAE: National Cancer Institute Common Toxicity Criteria for Adverse Events
PFS: Progression free survival
G-CSF: Granulocyte-colony stimulating factor
rhTPO: Recombinant human thrombopoietin
NIPS: Neoadjuvant intraperitoneal and systemic chemotherapy
DTX: Docetaxel
CRS: Cytoreductive surgery
